# Supplementary material for: Asynchronous magnetic resonance elastography: Shear wave speed reconstruction using noise correlation of incoherent waves
Source: Magn Reson Med. 2022 Oct 27;89(3):990–1001. doi: 10.1002/mrm.29502 (PMC9792433; doi:10.1002/mrm.29502)
Supplement: Supplementary file 1 — DATA S1 MATLAB code used to generate the numerical simulations of Figure 1 [file MRM-89-990-s002.zip › k-Wave/helpfiles/getWin.html]

getWin :: Functions (k-Wave)


# getWin

Return a frequency domain windowing function.

## Syntax

```
win = getWin(N, type)
win = getWin(N, type, ...)
[win, cg] = getWin(N, type)
[win, cg] = getWin(N, type, ...)
```

## Description

`getWin` returns a 1D, 2D, or 3D frequency domain window of the specified type of the given dimensions. By default, higher dimensional windows are created using the outer product. The windows can alternatively be created using rotation by setting the optional input `'Rotation'` to `true`. The coherent gain of the window can also be returned. Several examples are given below.

```
getWin(32, 'Hanning', 'Plot', true);
```

```
getWin(32, 'Hanning', 'Plot', true, 'Symmetric', false);
```

```
getWin([32, 32], 'Gaussian', 'Plot', true);
```

```
getWin([30, 40], 'Gaussian', 'Plot', true, 'Rotation', true);
```

## Inputs

|  |  |
| --- | --- |
| `N` | number of samples, use  `N = Nx` for 1D `N = [Nx Ny]` for 2D `N = [Nx Ny Nz]` for 3D |
| `type` | window type. Supported values are  `'Bartlett'`  `'Bartlett-Hanning'  'Blackman'  'Blackman-Harris'  'Blackman-Nuttall'  'Cosine'  'Flattop'  'Gaussian'  'HalfBand'  'Hamming'  'Hanning'  'Kaiser'  'Lanczos'  'Nuttall'  'Rectangular'  'Triangular'  'Tukey'` |

## Optional Inputs

Optional 'string', value pairs that may be used to modify the default computational settings.

| Input | Valid Settings | Default | Description |
| --- | --- | --- | --- |
| `'Plot'` | *(Boolean scalar)* | `false` | Boolean controlling whether the window is displayed. |
| `'Param'` | *(numeric scalar)* | `0.16 ('Blackman')` `0.5 ('Gaussian')` `3 ('Kaiser')` `0.5 ('Tukey')` | Control parameter for the Blackman, Gaussian, Kaiser, and Tukey windows. |
| `'Rotation'` | *(Boolean scalar)* | `false` | Boolean controlling whether 2D and 3D windows are created via rotation or the outer product. Windows created via rotation will have edge values outside the window radius set to zero. |
| `'Symmetric'` | *(Boolean)* | `true` | Boolean controlling whether the window is symmetrical. If set to false, a window of length N + 1 is created and the first N points are returned. For 2D and 3D windows, `'Symmetric'` can be defined as a vector defining the symmetry in each matrix dimension. |
| `'Square'` | *(Boolean scalar)* | `false` | Boolean controlling whether the window is forced to be square. If set to true and Nx and Nz are not equal, the window is created using the smaller variable, and then padded with zeros. |

## Outputs

|  |  |
| --- | --- |
| `win` | window |
| `cg` | coherent gain of the window |

## Examples

- Image Reconstruction With Compensation For Acoustic Absorption
- Source Smoothing
